# Nitrogen catabolite repressible GAP1 promoter, a new tool for efficient recombinant protein production in *S. cerevisiae*

**DOI:** 10.1186/1475-2859-12-129

**Published:** 2013-12-26

**Authors:** Fabien Debailleul, Cataldo Trubbia, Nancy Frederickx, Elsa Lauwers, Ahmad Merhi, Jean-Marie Ruysschaert, Bruno André, Cédric Govaerts

**Affiliations:** 1 S.F.M.B., Université Libre de Bruxelles, Blvd. du Triomphe, Bâtiment BC, local 1C4.208, B-1050 Bruxelles, Belgium; 2 Lab Physiologie Moléculaire de la Cellule, Université Libre de Bruxelles, IBMM, rue des Pr. Jeener et Brachet, 12, 6041 Gosselies, Belgium

**Keywords:** Saccharomyces cerevisiae, Protein expression, Protein purification, Heterologous expression

## Abstract

**Background:**

Decades of work requiring heterologous expression of eukaryotic proteins have shown that no expression system can be considered as the panacea and the appropriate expression strategy is often protein-dependent. In a large number of cases, yeasts have proven to be reliable organisms for heterologous protein expression by combining eukaryotic cellular organization with the ease of use of simpler microorganisms.

**Results:**

During this work, a novel promoter system based on the nitrogen catabolite regulation has been developed to produce the general amino acid permease (Gap1) in its natural host, the yeast *Saccharomyces cerevisiae.* A simple purification protocol was also established that allows to purify milligrams of Gap1 from cells cultivated in a five liters bio-reactor. In order to test the ability of the system to be used for expression of other proteins, the yeast specific transporter of γ-aminobutyric acid (Uga4), a human vesicular transporter of glutamate (Vglut1) and a small secreted glycoprotein (MD-2) were also expressed using the nitrogen catabolite regulation. All proteins were fused to GFP and their presence and localization were confirmed by western blot analysis and fluorescence microscopy.

**Conclusions:**

Our work shows that the nitrogen catabolite repressible GAP1 promoter can be used to obtain high levels of recombinant protein while allowing for large biomass production in *S. cerevisiae*. This approach can be used to express membrane and soluble proteins from higher eukaryotes (from yeast to human). Therefore, this system stands as a promising alternative to commonly used expression procedure in yeasts.

## Background

Genes coding for membrane proteins account for 20% to 30% of all Open Reading Frames present in sequenced genomes [1] and membrane proteins represent more than 60% of the drug targets but about only 1% of known protein structures [2,3]. Expression, purification and crystallization of these proteins remain a difficult task to achieve and are very protein-dependent. Importantly, membrane proteins of known structure are mostly of prokaryotic origin, probably due to the difficulties associated with the expression of eukaryotic proteins in scalable systems. Yeasts, and *Saccharomyces cerevisiae* in particular, have notably proven to be a reliable system of expression for both endogenous and heterologous eukaryotic proteins [4-7]. The extensive knowledge in *S. cerevisiae* regulation and synthetic pathways, combined with the reduced cost associated with this type of organism, allows to tailor adaptive protocols for the expression of proteins [8,9].

As any other eukaryotic organism, yeasts possess a typical internal organization with membrane-delimited organelles. Membrane proteins destined to the plasma membrane traffic through the endoplasmic reticulum and Golgi apparatus and eventually undergo post-translational modifications similar to those occurring in higher eukaryotes, although proteins tend to be over-glycosylated when expressed in yeasts [10,11]. As unicellular and simple organisms, yeasts are very easy to grow and cultures are cost-effective. *S. cerevisiae* in particular has been used and studied for many years and a wide range of mutants and deletion strains are available. Moreover, a large number of expression vectors are available for protein production in *S. cerevisiae* and transformation-associated *in vivo* recombination in these vectors allows to easily test various plasmid constructs (harboring alternative gene promoters, tags, linkers, and eventually including mutations in the genes of interest). Although the biomass obtained from expressing cells can be lower than for other yeasts (such as *Pichia pastoris*), the relative amount of protein of interest versus the total biomass can result in better purification yield and purity [12].

Here we developed a novel promoter system in *S. cerevisiae* aiming at producing large quantities of recombinant membrane or soluble proteins. We originally designed the system for Gap1, the general amino acid permease of *S. cerevisiae*[13,14]. Gap1 is a member of the amino acid-polyamine-organocation (APC) superfamily. It can mediate uptake of all protein amino acids, several non-protein amino acids (e.g., ornithine, citrulline, gamma-aminobutyic acid, beta-alanine) and toxic analogs. Gap1 shows very high affinity for most of its natural substrates, with apparent Km values in the micromolar range [13]. These properties are well suited to the physiological role of Gap1, which is synthesized and most active under conditions of poor nitrogen supply (e.g. proline, urea, low ammonium, etc.). The role of Gap1 under these conditions is to scavenge external amino acids in order to be used as nitrogen sources or directly as building blocks for protein synthesis. Transcription of the GAP1 gene is promoted by two GATA-family factors, Gln3 and Gat1, which are mostly active when the nitrogen supply conditions are cell-growth limiting. If cells shift to more favorable nitrogen supply conditions, the Gln3 and Gat1 factors are inhibited by the mechanisms of Nitrogen Catabolite Repression (NCR), leading to a strong decrease in *GAP1*’s expression [15].

This conditional transcription was used to design an inducible promoter system in *S. cerevisiae* where first, biomass is accumulated while expression of the target protein is strongly repressed and, secondly, expression is triggered following an appropriate medium change. Practically, cells were cultivated in a bio-reactor on a rich medium (based on yeast extract and Bactopeptone) containing large quantities of amino acids and other biosynthetic precursors (thus providing optimal nitrogen supply conditions) and glucose as carbon source. Under these conditions, yeasts follow a two-phase growth [16]: they first metabolize glucose by fermentation (even in the presence of oxygen) and then switch to a fully respiratory metabolism. This switch, also referred to as diauxic switch, can be detected by measuring the oxygen concentration in the medium. After biomass accumulation until the diauxic switch, expression of the target protein is triggered by exchanging the rich medium for a defined medium containing a secondary (poor) source of nitrogen.

Based on our expression results with Gap1, we subsequently extended this expression system to three unrelated proteins: the yeast specific transporter of γ-aminobutyric acid (GABA) (Uga4, [17]), a human vesicular transporter of glutamate (Vglut1, [18,19]) and a small human secreted glycoprotein (MD-2, [20,21]). These proteins were expressed under the regulation of the Gap1 promoter and their expression was tested by immunoblotting and GFP fluorescence localization.

## Results and discussion

### Comparison between constitutive and induced productions of Gap1

**Figure 1:**
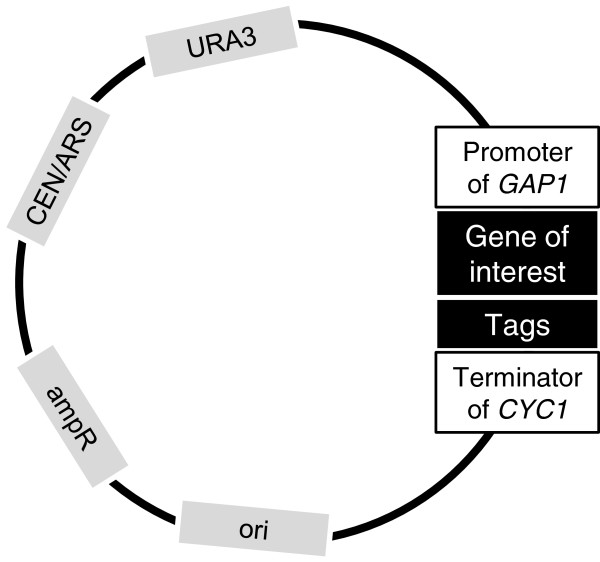
**Schematic representation of the vectors used during this work.** They all derived from pRS416 and contain the same features: a yeast selection marker (*URA3*), an origin of replication for propagation in yeast (CEN/ARS), a marker of selection and an origin of replication for bacteria (ori and ampicillin resistance). All constructs were obtained by *in vivo* recombination.

In order to assess the ability of our expression protocol to provide large quantities of the target protein, we selected a mutant version of Gap1, Gap1^9KR^, where the first 9 lysines present in the cytosolic amino-terminal part are mutated into arginine in order to protect the protein against ubiquitylation, thus making it resistant to endocytosis and subsequent degradation without affecting its activity [22]. The sequence of *GAP1–9KR* was fused at its C-terminus to a double affinity tag made of the Gluthatione-S-Transferase and a short sequence of 6 histidines (*GAP1-GST-6HIS*). The tags are separated from the protein by a poly-Gly-Ala linker (GA_5_) and a cleavage site for the HRV 3C protease (LEVLFQGP). The double tag was used to facilitate the purification of the protein and avoid a negative effect of the histidine-tag on the activity of the protein *in vivo* as detected during preliminary experiments (see Additional file 1: Figure S1). All plasmids were obtained by Transformation-Associated Recombination (TAR) cloning. This technique allows to directly introduce PCR fragments into linearized plasmids by homologous recombination in yeast [23]. All expression vectors used during this study derived from pRS416 [24], a low copy (CEN/ARS) plasmid carrying a *URA3* selection marker (Figure 1). All experiments were carried out in a *ura3* mutant derived from the Σ1278b wild-type strain where the endogenous *GAP1* gene was deleted.

The expression of *GAP1-GST-6HIS* was tested under the regulation of 3 promoters: the constitutive promoter of the plasma membrane H^+^-ATPase gene (P_PMA1_), the promoter of the galactokinase GAL1 gene (P_GAL1_) which is regulated by the carbon source and the natural promoter (P_GAP1_), an inductive promoter subject to NCR. P_PMA1_ and P_GAL1_ have been already used to express membrane proteins [12,25,26] but P_GAP1_ has not. Genes under the regulation of P_PMA1_ are constitutively expressed regardless of the medium composition. Conversely, genes under the regulation of P_GAL1_ or P_GAP1_ are only produced when a minimal medium is used in order to impose a specific carbon source for P_GAL1_ (galactose) or a specific nitrogen source for P_GAP1_ (proline).

**Figure 2:**
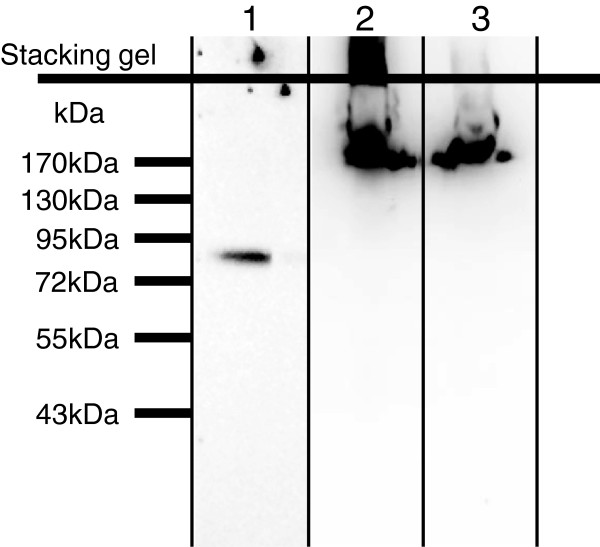
**Western blot showing the expression of Gap1-GST-6his developed with an antibody directed against penta-histidines.** The gene is expressed under the regulation of its natural promoter (1, minimal medium) or under the regulation of the promoter of *PMA1* (2, minimal medium; 3, rich medium). The same amount of total protein is loaded on each lanes. The stacking gel was also transferred and its limit is indicated.

The *GAL1* promoter was tested during the early stages of development of the expression strategy but was discarded due to a lower activity measured *in vivo* (compared to the protein expressed with the wild type promoter) and a severely reduced generation time on inductive medium (from an expected 90 minutes doubling time at 30°C down to more than 4 hours). Expression under the regulation of the natural and *PMA1* promoters was tested by western blot analysis on minimal (inductive) medium for P_GAP1_ and on both minimal and rich medium for P_PMA1_. Gel electrophoresis analysis showed that produced Gap1 typically appears as a double or blurred band (due to phosphorylation [27]) at a size slightly smaller than expected (Gap1-Gst-6His, 849 amino acids, 94.2 kDa). As shown in Figure 2, Gap1 expressed under its natural promoter was detected at the proper molecular weight but migrated at a much higher size when the protein was expressed using P_PMA1_. In addition, the generation time of the cells, already affected when the protein was expressed under the regulation of P_GAP1_ (from an expected 80 minutes doubling time at 30°C down to approximately 2 hours), was severely reduced by the constitutive expression under P_PMA1_ (more than 4 hours) when cultivated on minimal medium. These findings led us to establish a specific protocol for expression under the regulation of the natural *GAP1* promoter.

### High level production of Gap1

**Figure 3:**
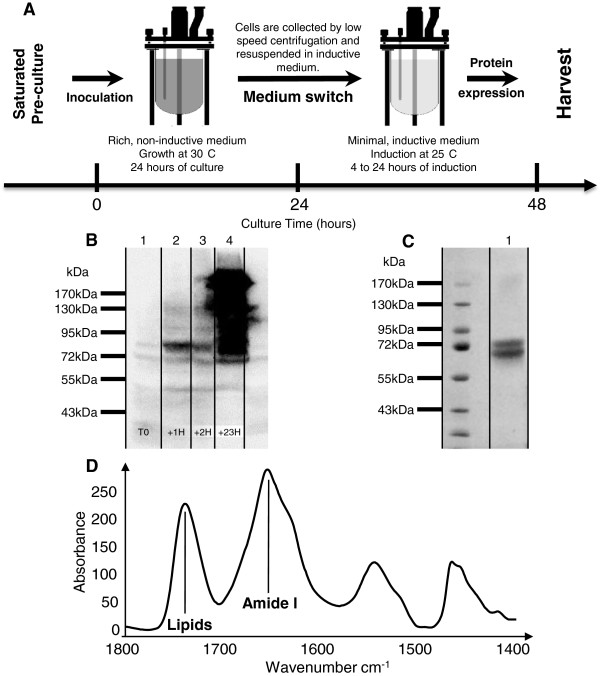
**Expression, purification and characterization of Gap1expressed using the nitrogen catabolite repression promoter system. A)** Schematic representation of the medium switch protocol. **B)** Expression control of Gap1 by western blot revealed by an antibody against penta-histidines. Samples were taken just before the switch to the inductive medium (1), one hour after medium switch (2), 2 hours (3) and 23 hours just before harvesting the cells (4). **C)** Purity control by SDS-PAGE of Gap1 after purification using affinity chromatography. The protein is revealed by Coomassie staining. **D)** Infrared spectrum of reconstituted Gap1 in yeast lipid extract, peak of C = O bounds of lipids and proteins are highlighted.

We decided to take advantage of the inductive nature of P_GAP1_ to accumulate biomass during a first phase before switching to an inductive (minimal proline) medium and thus trigger protein production. Practically, a saturated pre-culture grown on minimal proline medium was diluted (100-fold) in a five-liter bio-reactor containing rich medium. Through simple regulation loops, the concentration of dissolved oxygen was kept at least at 40% of its original value by simply controlling the stirring speed while the pH was kept above 5.5 to avoid excessive acidification of the medium. After 24 hours, a drop in the oxygen consumption indicated that the cells undergo a diauxic shift toward a fully respiratory metabolism as the glucose present as carbon source is no longer present in sufficient quantity to sustain fermentative growth. The cells were centrifuged and resuspended in fresh, minimal proline medium and the culture was carried on for 24 hours in the bio-reactor. Cells were harvested after 24 hours of induction. Typical yields per liter of culture were: about 4 grams of wet cells for agitated flasks, 8 grams for bio-reactor with minimal, inductive medium and more than 40 grams of wet cells with the medium switch protocol. Thus, using a small 5 L fermenter, about 200 grams of cells can be produced per batch of culture. As expected, Gap1 expression was strongly repressed during the growth on rich medium (Figure 3B.1), and a strong induction of production was observed directly after medium switch and during all the culture on minimal medium (Figure 3B.2–4).

### Purification of Gap1

A detailed purification protocol is described in “Material and Methods” but the determinant factors are discussed in this section. Yeasts are surrounded by a thick cell wall that can be removed by mechanical disruption (glass beads or high pressure homogenizer) or by enzymatic digestion. The latter being quite expensive for large amount of cells, the mechanical approaches were preferred. In particular, the high pressure homogenization (Avestin EmulsiFlex-C3) was chosen for its reproducibility, efficiency and ease of use, as compared to cell disruption using glass beads. As the technique tends to break up vacuoles and causes the release of proteases, a specific cocktail of protease inhibitors (leupeptin, chymostatin, pepstatin, PMSF, and EDTA to inactive metal-dependent proteases) was used to avoid target proteolysis. Typically, 40 to 50 grams of wet cells (corresponding to 1/4 of a bio-reactor) were resuspended in 200 mL of lysis buffer and disrupted in the presence of protease inhibitors. Cells lysate was cleared by low speed centrifugation at 3,000 g and membranes were pelleted at 120,000 g. Solubilization is a crucial step for membrane protein purification. A wide variety of detergents are available to extract the target protein from the membrane and to maintain it in solution while avoiding any irreversible unfolding during the purification. In order to find the most suitable detergent for Gap1 purification, several commonly used detergents were tested: zwitterionic long chain fos-cholines FC14 and FC16, glycosylated short chain octyl- and nonyl-glucosides OG and NG, and glycosylated long chain decyl- and dodecyl- maltopyranoside DM and DDM. Surprisingly, only DDM was able to solubilize and keep the protein migration profile unaffected. All other detergent treatments led to protein degradation and alteration in migration profiles. Pelleted membranes were resuspended in the same buffer but without EDTA and solubilized by addition of DDM (2% final). Insolubilized membranes were pelleted at 120,000 g and the supernatant applied on pre-equilibrated Ni-NTA resin. A critical background concentration of 10 mM of imidazole was used during the purification to avoid non-specific binding to the resin. Based on the SDS-PAGE analysis reported in Figure 3C.1, Gap1 eluted from Ni-NTA resin was almost pure. About 1 milligram of Gap1 was obtained for 1 liter of culture. The purified sample was also subjected to size-exclusion chromatography to measure the monodispersity of the protein (see Additional file 2: Figure S2). We observe a major peak corresponding to the monomeric form of the protein embedded in a detergent micelle. In addition, significant amounts of multimeric forms are present, indicating that further improvements in the purification protocol may be required for applications requiring strictly monodisperse protein (i.e. crystallography). On SDS-PAGE, the purified Gap1 appears as a defined double band and no multimeric forms appear after purification. These forms can be observed in the total protein extract but are either transient or not purified. The double band was previously reported to be due to the phosphorylation of the protein [27]. After alkaline phosphatase treatment on our purified sample, we observe the disappearance of the upper band, confirming that the doublet is due to phosphorylation (see Additional file 3: Figure S3). To assess the integrity of the protein, we have reconstituted Gap1 in a commercial lipid extract of *S. cerevisiae* and measured the sample by infrared spectroscopy. Spectra obtained by attenuated total reflection infrared spectroscopy (ATR-FTIR) contain bands of absorption of all chemical bounds present in the sample at a wavenumber depending on their chemical environment. This allows to see signature absorption bands of chemical bounds of lipids and proteins and to evaluate secondary structure content of the sample. As can be seen in Figure 3D, our sample contains both signature bands of lipid and protein, and the band of protein has its maximum of absorption at 1655 cm^−1^, which is typical of α-helix proteins, as expected of a member of the APC family [28,29]. To our knowledge, it is the first reported expression, purification and reconstitution of Gap1.

### Extension of the production protocol to other proteins Table 1

**Table 1:** Summary of the proteins expressed during this work

**Protein**	**Natural host**	**Native localization**	**Function**	**Expected size (kDa)**	**Reference**
Gap1	S. cerevisiae	Plasma membrane	General amino acid transporter	65.9	[14]
N-ter Gap1	S. cerevisiae	Cytosol	Cytosolic N-terminus part of Gap1	9.4	This publication
Uga4	S. cerevisiae	Plasma membrane	GABA-specific permease	61.9	[17]
Vglut1	Human	Vesicles	Glutamate transporter	61.6	[18,19]
MD-2	Human	Extracellular periphery	Lipid-binding protein, LPS co-receptor	18.3	[20,21]

**Figure 4:**
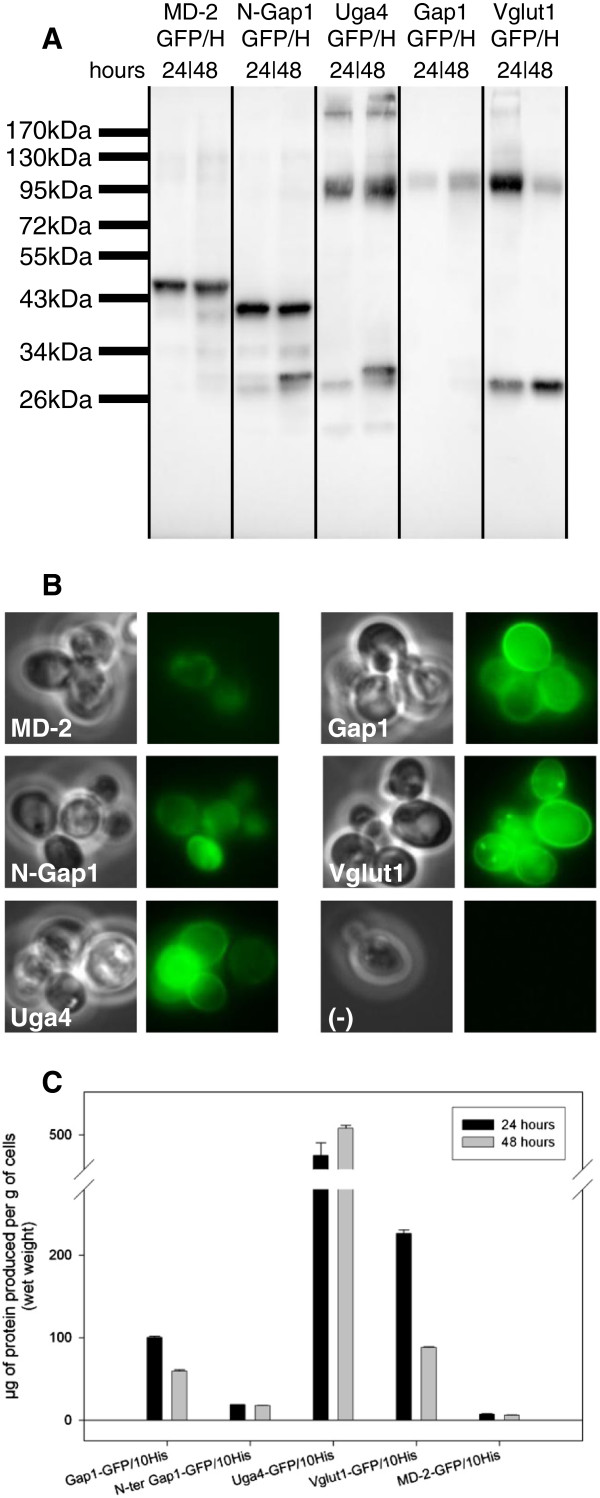
**Quantification and localization of MD-2, N-Gap1, Uga4, Gap1 and Vglut1 expressed using the nitrogen catabolite repression promoter system. A)** Expression control by western blot done on total cellular extract after 24 and 48 hours of culture on inductive medium. The same amount of total protein (normalized by OD measurement) was loaded on each lanes. Proteins are revealed by an antibody against GFP. Expected sizes for the GFP-tagged proteins: MD2 (47.6 kDa), N-ter Gap1 (38.7 kDa), Uga4 (91.2 kDa), Gap1 (95.2 kDa), Vglut1 (90.9 kDa). **B)***In vivo* fluorescence of expressing cells cultivated 24 hours on inductive medium. Untransformed cells have been used as negative control (−). All fluorescence pictures were taken using the same exposure time. **C)** Quantification of expression by GFP fluorescence measurements. Cells cultivated for 24 and 48 hours on inductive medium were submitted to GFP fluorescence measurements. Signal from non-induced cells was used for auto-fluorescence background subtraction. Fluorescence intensity were compared to a standard curve established using purified GFP. The quantification was calculated per gram of wet weight cells, corresponding to ~25 ml of fermenter culture at OD_660_ 40.

Given the promising results obtained for the Gap1 expression, the ability of the expression vector containing P_GAP1_ to express other proteins was also studied. We introduced coding sequences for another yeast plasma-membrane protein, Uga4, the specific transporter of γ-aminobutyric acid (GABA) [17]. We also included two human proteins, Vglut1, a vesicular transporter of glutamate [18,19] already expressed in higher eukaryote systems [30] and MD-2, a small secreted glycoprotein [20,21], already expressed in *P. pastoris*[31] and insect cells [32] although, here, expression is targeted to the cytoplasm following removal the 16–amino acid secretion signal from the N terminus. Finally, the N-terminus cytosolic part of Gap1 that has been reported to be implicated in the protein regulation [33] was expressed separately as a soluble domain. In order to improve the expression of *VGLUT1*, we obtained a codon-optimized synthetic gene. All proteins were tagged at their C-terminus with a GFP/decahistidines tag separated from the protein by a poly-Gly-Ala linker (GA_5_) and a cleavage site for the HRV 3C protease. As a control, Gap1 was also expressed with the same tags. GFP is a very useful reporter for protein expression for two reasons. First, it was shown in *Escherichia coli* that, when placed at the C-terminus of another protein, GFP-folding depends on prior folding of the upstream protein, i.e. fluorescence is observed if the protein of the N-terminal side is properly folded [34,35]. Second, in *S. cerevisiae*, misfolded membrane proteins are either retained in the endoplasmic reticulum (ER) until they are folded correctly or dislocated from the ER and degraded. In both cases, observing GFP localization using fluorescence microscopy will allow us to verify the subcellular targeting of the expressed protein and provides a good indication of correct folding. Initial expression tests on inductive medium, evaluated by *in vivo* fluorescence and western blot, revealed a poor expression for all targets. Based on previous literature data [26], the expression protocol was optimized by switching the expression temperature to 25°C and adding 10% glycerol to the medium as chemical chaperone. These two changes dramatically increased the expression without affecting the overall biomass production. The modifications were also tested in bio-reactor for the expression of Gap1, where the lower temperature and glycerol were used only during the inductive phase without affecting the biomass yield or the protein expression. As can be seen in Figure 4B, Gap1 and Uga4 are localized mainly at the plasma membrane; Vglut1 is also located at the plasma membrane but also in dotted structures inside the cell, finally MD-2 and the N-terminus part of Gap1 are located in the cytosol. These correspond to the expected localizations of each protein and the fluorescence intensity is in the same range for all proteins, with a slightly weaker signal for MD-2. The western blot (Figure 4A) revealed by an antibody against GFP confirms that all proteins are produced but differences in the migration profile between 24 and 48 hours indicate that kinetic of expression is protein-specific and must be optimized for every target. Some low-weight bands are present, for example in the case of Vglut1, indicating that partial degradation may occur. This could be circumvented by lowering the temperature or diminishing the induction time but, as can be seen by fluorescence, a large fraction of target proteins are fluorescent and properly localized. For Uga4 some highmers are present on the western blot. We note however that for every case the majority of the protein is present as a single band migrating at the expected size, in sharp contrast to what we observed for Gap1 under the regulation of P_PMA1_ (Figure 2). Adequate localization shown by GFP-fluorescence microscopy further suggest that most of the protein is expected to be properly expressed.

In order to quantify the level of expression of each protein, a whole cell fluorescence measurement was done on the cells cultivated for 24 hours. The measurement was done according to previous publication [36] and Table 2 provides an estimation of the total quantity of protein produced per gram of wet cell weight which corresponds to ~25 ml of fermenter culture at OD_660_ 40.

**Table 2:** Protein quantification by whole cell fluorescence measurements

**Protein**	**Expected size (kDa)**	Fluorescence *		**Estimated quantity**	
		**(Arbitrary units)**		(μg per g of cells**)	
		**24 h.**	**48 h.**	**24 h.**	**48 h.**
Gap1-GFP/10His	95.2	3325 ± 49	1971 ± 54	100	60
N-ter Gap1-GFP/10His	38.7	4424 ± 18	733 ± 38	19	18
Uga4-GFP/10His	91.2	16801 ± 514	17914 ± 123	476	508
Vglut1-GFP/10His	90.9	8032 ± 161	3129 ± 32	226	88
MD-2-GFP/10His	47.6	865 ± 113	4130 ± 66	7	6

*Background signal from non induced cells was subtracted.

**Wet weight.

## Conclusions

During this study, we have established a simple and efficient system to produce and purify milligrams of the general amino transporter of yeast *S. cerevisiae,* such expression and purification has never been reported so far. This system combines high yield, ease of use and low cost of production. To our knowledge, this is also the first time that the NCR is used to control protein production. This efficient and tightly regulated pathway coupled with fairly simple bio-reactor techniques has led us to this new system for protein expression. Such quantities of purified Gap1 allow proceeding with *in vitro* experiments and envisaging structural biology techniques. Although many studies have deciphered the regulation of the protein, both at the transcription and the protein level [13,37,38], little is known about the transport mechanism and the substrate recognition [39,40]. We expect that this production tool will facilitate *in vitro* studies and overall understanding of the protein. From this perspective, we have successfully reconstituted the protein in liposomes made of yeast lipids extract which pave the way to transport assays and structural biology experiments.

From a methodological perspective, by combining our system with the well-established GFP reporter [12,41], we have shown that it can be used to express human proteins, both soluble and inserted in the membrane. Compared to the promoter of *PMA1*, an inducible promoter such as the promoter of Gap1 seems more relevant for protein overexpression: constitutive overexpression slows cell growth dramatically and in our hands, leads to proteins aggregation. Renewing the medium before expression requires some additional manipulation compared to constitutive expression but allows reaching higher biomass per liter of culture (40 grams versus 8 grams per liter) and can be useful for secreted proteins that are not stable at low pH or are sensitive to yeast byproducts (e.g. ethanol). As Gap1 is not an essential gene and the results presented here were obtained with a centromeric (low copy) plasmid, direct genome integration can be used to avoid plasmid instability, a recurring problem in yeast protein expression, without affecting the number of copy of the gene.

Taken together, our results show that NCR in general and the Gap1 promoter in particular can be used to create a robust, cost-effective and high-throughput expression system in *S. cerevisiae* that can be used when specific requirements are needed such as a eukaryotic system, membrane integration or difficult protein secretion.

## Methods

### Strains, plasmids and media

All expression vectors were tested in a Σ1278b *gap1Δ ura3* background [42]. Cells were either grown in a rich medium (1%(w/v) yeast extract, 1%(w/v) bactopeptone, 2%(w/v) glucose) or in a defined medium (MgSO_4_ 3 mM, KH_2_PO_4_ 7 mM, CaCl_2_.2H_2_O 3 mM, NaCl 9 mM, K_2_SO_4_ 6 mM, citric acid 50 mM, KOH 160 mM, proline 9 mM, glucose 3%(w/v), pH 6.1, traces of metals and vitamins). Cultures were grown at 30°C and expression induced at 30 or (where specified) 25°C. During expression phases, the medium was supplemented with 10% glycerol as a chemical chaperone [26]. Medium switches were performed by centrifugation at moderate speed (7,000 g during 15 min) and resuspension in inductive medium. Cells were grown either in agitated flasks or in bio-reactor (Sartorius BIOSTAT® A+).

Genes were inserted in a plasmid containing URA3 (yeast selection marker), CEN/ARS (yeast origin of replication), ampR (bacteria selection marker) and ori (bacteria origin of replication). Expression vectors were obtained by in vivo recombination in yeast [23]: genes were amplified by PCR using specific oligonucleotide primers containing 40 bases corresponding to the target vector at their 5′ extremities. The vector (already containing the GAP1 promoter and the CYC1 terminator obtained the same way) was linearized using a specific restriction enzyme (*AgeI*). Both the linearized vector and the PCR products were transformed in yeast using the lithium acetate technique [43]. Transformants were plated on defined medium supplemented with 1%(m/v) agarose. Colonies generally appeared after 2 days at 30°C. Two to four colonies were cultivated on selective medium and their DNA was extracted using phenol/chloroform technique [44]. The extracted DNA was transformed in bacteria in order to produce sufficient amounts of DNA at required purity to perform sequencing. Confirmed DNA was transformed in expression strain and transformants were saved at −80°C.

### Activity assay

Gap1 activity was determined by measuring the initial rate of uptake of ^14^C- labelled citrulline (20 mM), a specific substrate of the permease [45]. At each expression time point (0, 1, 2 and 4 hours), a known quantity of ^14^C-citrulline was added to the cells. A small amount of cells was collected and washed after 30, 60 and 90 seconds and the radioactivity was counted for each sample. A linear regression was operated on the three values and the slope was extracted. This slope was normalized by the total quantity of protein and the activity of ^14^C-citrulline. The resultant protein’s activity was expressed in nmol of ^14^C-citrulline per minutes and milligrams of proteins. All measurements were done three times and their consistency was verified.

### Bio-reactor cultures

All cultures were grown at 30°C unless otherwise specified. A saturated pre-culture done on minimal proline medium was diluted (100-fold) in a five liter bio-reactor containing rich medium. The bio-reactor (Sartorius Biostat A+) is equipped with a pH probe, an oxygen probe, a level/foam detector, an air injector, a four-way injector, a rotor, an external heating blanket and cooler devices for both exhaust gas and internal medium. All operations were controlled by an external computer and a dedicated software (BIOSTAT Aplus PC Panel μDCU). Foam formation was prevented by addition of antifoam and pH was maintained at 5.5 by addition of 5 M KOH during the process. Oxygen concentration inside the medium was kept above 40% of its initial concentration by increasing the stirring speed. After 24 h, a drop in oxygen consumption was observed and the medium was exchanged by centrifugation (15 min, 7,500 g, Avanti™ J-20 XPI Centrifuge 6 L, Beckman Coulter) with fresh minimal medium (containing 0.3% proline and 3% glucose). Depending on the experiment, minimal medium was supplemented with 10% glycerol to increase protein stability and/or the temperature was lowered to 25°C. The induction time varied from 2 to 24 hours. Cells were then harvested by centrifugation (15 min, 7,500 g) and washed twice in decreasing amount of Tris–HCl (10 mM, pH7.5). After the last centrifugation, cells were frozen at −80°C.

### Purification of Gap1

Yeast were resuspended in 5 volumes of lysis buffer (40–50 grams of cells in 200 mL final) containing Tris–HCl pH 7.5 100 mM, NaCl 150 mM, glycerol 10%(m/v), beta-mercapto-ethanol 20 mM, EDTA 10 mM, leupeptin 10 μg/mL, pepstatin 10 μg/mL, chymostatin 2.5 μg/mL and PMSF (phenylmethanesulfonylfluoride) 2.5 mg/mL. Cells were disrupted by homogenization using an Avestin EmulsiFlex C3 (1 passage at low pressure and 3 passages at more than 25,000 psi). Cell debris were removed by low speed centrifugation (15 min at 3,000 g). The supernatant was ultracentrifuged (1 hour at 125,000 g) to pellet the membranes. Membranes were resupended in 50 mL purification buffer containing Tris–HCl pH 7.5 100 mM, NaCl 150 mM, glycerol 10%(m/v), beta-mercapto-ethanol 1 mM, tris(2-carboxyethyl)phosphine (TCEP) 100 μM. Solubilization was achieved by incubation under slow agitation at 4°C with 2%(m/v) DDM (n-dodecyl-beta-D-maltopyranoside). The fraction resistant to detergent was removed by another ultracentrifugation step and the supernatant was applied on Ni-NTA resin. Imidazole (10 mM final) was added to avoid unspecific binding on the column and high contamination of the final sample. Proteins were incubated 1 hour at 4°C with the resin under slow agitation. The resin was washed with 5 Column Volumes (CV) of purification buffer containing 10 mM imidazole and 2x CV containing 20 mM imidazole. Gap1 was eluted with 250 mM imidazole by fraction of 500 μL (5 to 7 CV). The most concentrated fractions were pooled together and tested by SDS-PAGE. Size exclusion chromatography was used to control the dispersity of the purified Gap1. The pooled fractions were loaded on a size exclusion column (SDX-200 10/300GL) and the protein was detected by UV absorption. Molecular weight standards (BioRad) were used to estimate the apparent mass. The phosphorylation was assayed by treating the purified protein with alkaline phosphatase (Roche) according to the manufacturer instructions and Gap1 was revealed using a polyclonal antibody raised against Gap1 [27].

### Reconstitution of Gap1 and infrared spectroscopy

A dried film of yeast lipid extract (2 mg) (Avanti Polar Lipids, Inc. - Yeast Total Lipid Extract) was obtained by evaporation of chloroform under a flow of nitrogen, followed by overnight drying under vacuum. Liposomes were prepared by sonication of the lipid film for 3 × 1 min on a 250 W Vibra Cell Sonifier in 300 μL Tris–HCl pH 7.5 20 mM, NaCl 75 mM. The purified Gap1 in the presence of 0.5% DDM was mixed with liposomes at a protein to lipid ratio of 1:50 w/w and incubated for 30 min at 4°C under gentle agitation. The detergent was then removed by absorption on SM2 Bio-Beads (four incubations of respectively: one hour, overnight and twice one hour under agitation in the presence of 40 mg Bio-Beads).

Infrared spectroscopy spectra were taken using Attenuated Total Reflection-Fourier Transform Infrared spectroscopy (ATR-FTIR) [46]. ATR-FTIR spectra were recorded at room temperature on a Equinox 55 infrared spectrophotometer (Bruker Optics) equipped with a Golden Gate reflectance accessory (Specac) at a nominal resolution of 2 cm^−1^ and encoded every 1 cm^−1^. The spectrophotometer was continuously purged with air dried on a FTIR purge gas generator 75 – 62 Balston (Maidstone, UK) at a flow rate of 5.8 L/min. The internal reflection element was a diamond crystal (2 mm × 2 mm) with an aperture angle of 45° that yielded a single internal reflection. Samples were prepared by spreading 2 μl of the proteoliposomes suspension on the diamond crystal surface and by removing the excess water under nitrogen flow. The signal from the atmospheric water was subtracted as described by Goormaghtigh and Ruysschaert [46]. The data presented here are the average of 128 spectra to improve the signal/noise ratio. Data were processed using “Kinetics”, an analysis program developed in our laboratory and running under MatLab (The MathWorks, Natick, MA).

### SDS-PAGE and immuno-blotting

Whole cell lysates (2 mL at OD_660_ 0.5) or purified proteins (30 μL at a protein concentration of 0.3 mg/mL) were analyzed by standard SDS-PAGE using polyacrylamide gels (Mini-PROTEAN® TGX™ Precast Gels, Bio-Rad Laboratories N.V.) followed either a Coomassie staining or a transfer to nitrocellulose membrane (Bio-Rad Laboratories N.V.) for immuno-blotting. Poly-histidines tags were detected using the QIAexpress® Anti-his Hrp Conjugate Kit (Qiagen) and GFP tags were detected using anti-GFP Rabbit IgG Polyclonal Antibody Fraction (Molecular Probes®). Proteins were visualized by electro-chemiluminescence using the Luminata™ Forte Western Hrp Substrate (Merck Millipore) and pictures were taken using the ECL-system ImageQuant 400® from GE Healthcare. Images were layered and cropped using Photoshop (Adobe).

### Fluorescence microscopy

GFP imaging was done using a microscope Axio Scope.A1 (Zeiss) with an objective 100X. 2 μL of a suspension of cells at OD_660_ 1 were spread on a glass slide and images were taken at room temperature without any kind of fixing. Images were taken using the Zeiss software (Axio Vision 4.8.2). Images were cropped using Photoshop (Adobe).

### GFP quantification

The expression of MD2, N-ter Gap1, Uga4, Gap1 and Vglut1 was evaluated by GFP quantification. A protocol analog to the one described by Drew, *et al.*[36] was used to quantify the expression. The GFP intensity of a known quantity of cells transformed with the corresponding expression vector and cultivated on inductive medium was measured and reported to the standard curve. A standard curve was established using successive dilutions of purified GFP, a 6-fold intensity factor was applied in order to take into account the fluorescence yield difference between wt GFP (used for the standard curve) vs. S65T GFP (present in the chimeric constructs) [47]. By taking into account the molecular weight of each proteins, we can estimate of the quantity produced for 40 grams of wet weight cells (a typical yield for one liter of culture in fermenter).

## Competing interests

The authors declare that they have no competing interests.

## Authors' contributions

FD: experimental design, molecular biology experiments, bio-reactor culture design, western blot experiments, data acquisition, manuscript redaction. CT: bio-reactor cultures, bio-reactor troubleshooting. NF: fluorescence expertise, data acquisition, molecular biology experiments. EL: molecular biology experiments. AM: biochemistry experiments. JMR: field expertise, manuscript review. BA: field expertise, yeast manipulation expertise, manuscript review. CG: study supervision, experiments coordination, field expertise, manuscript review. All authors read and approved the final manuscript.

## Supplementary Material

Additional file 1: Figure S1 Activity measurements of different tagged version of Gap1 expressed under the regulation of P_GAP1_. Gap1’s activity is determined by measuring the entrance of radio-labeled citrulline *in vivo*. Cells transformed with the expression vector containing GAP1 were grown on minimal medium - ammonium/glucose. At time 0, induction was triggered by switching to proline/glucose. Activity was measured at time 0 and after 1, 2 and 4 hours of induction. Click here for file

Additional file 2: Figure S2 Size exclusion profile of purified Gap1-GST/His. The most concentrated fractions of the affinity chromatography were pooled together and loaded on a size exclusion column (SDX-200 10/300GL). The protein is detected by UV absorption. The retention volumes of reference proteins are also indicated. Click here for file

Additional file 3: Figure S3 Alkaline phosphatase treatment of purified Gap1. Two independent batches of purified Gap1 were treated by alkaline phosphatase according to the manufacturer instructions. The protein was then resolved by electrophoresis and analyzed by immunoblotting. The protein was revealed by an antibody against Gap1 [27]. Click here for file
