# Supplementary material for: Nitrogen catabolite repressible GAP1 promoter, a new tool for efficient recombinant protein production in S. cerevisiae
Source: Microb Cell Fact. 2013 Dec 26;12:129. doi: 10.1186/1475-2859-12-129 (PMC3880969; doi:10.1186/1475-2859-12-129)
Supplement: Additional file 1: Figure S1 — Activity measurements of different tagged version of Gap1 expressed under the regulation of PGAP1. Gap1’s activity is determined by measuring the entrance of radio-labeled citrulline in vivo. Cells transformed with the expression vector containing GAP1 were grown on minimal medium - ammonium/glucose. At time 0, induction was triggered by switching to proline/glucose. Activity was measured at time 0 and after 1, 2 and 4 hours of induction. [file 1475-2859-12-129-S1.pptx]

## Slide 1
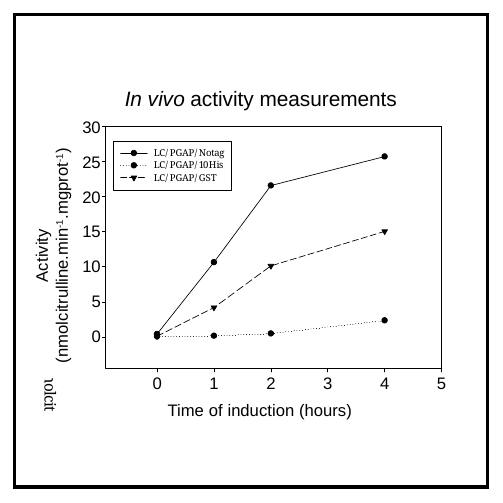

In vivo activity measurements
30
25
20
15
Activity
(nmolcitrulline.min-1.mgprot-1)
10
5
0
0
1
2
3
4
5
Time of induction (hours)
