# Supplementary figures and images for: Nitrogen catabolite repressible GAP1 promoter, a new tool for efficient recombinant protein production in S. cerevisiae
Source: Microb Cell Fact. 2013 Dec 26;12:129. doi: 10.1186/1475-2859-12-129 (PMC3880969; doi:10.1186/1475-2859-12-129)

## Slide 1
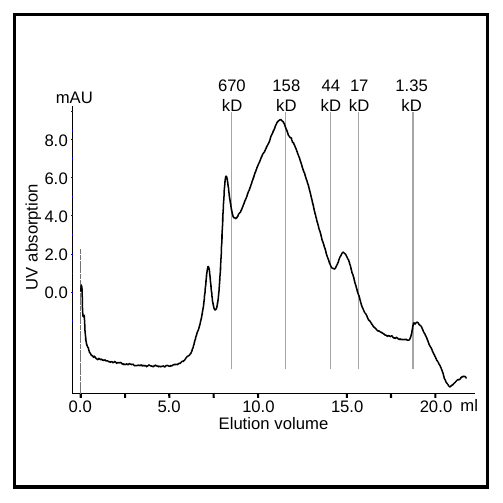

670
kD
158
kD
44
kD
17
kD
1.35
kD
mAU
8.0
6.0
4.0
UV absorption
2.0
0.0
ml
0.0
5.0
10.0
15.0
20.0
Elution volume

Supplement: Additional file 2: Figure S2 — Size exclusion profile of purified Gap1-GST/His. The most concentrated fractions of the affinity chromatography were pooled together and loaded on a size exclusion column (SDX-200 10/300GL). The protein is detected by UV absorption. The retention volumes of reference proteins are also indicated. [file 1475-2859-12-129-S2.pptx]

## Slide 1
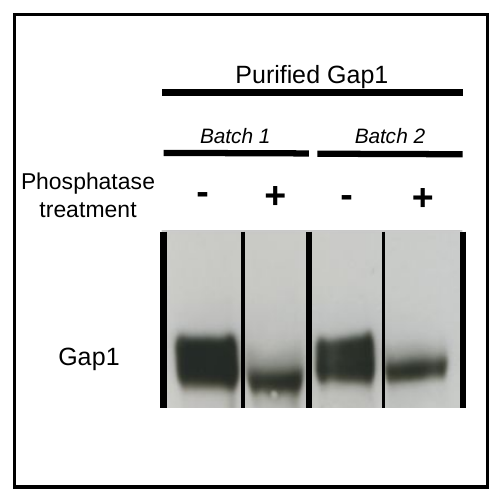

Purified Gap1
Batch 1
Batch 2
Phosphatase
treatment
-
-
+
+
Gap1

Supplement: Additional file 3: Figure S3 — Alkaline phosphatase treatment of purified Gap1. Two independent batches of purified Gap1 were treated by alkaline phosphatase according to the manufacturer instructions. The protein was then resolved by electrophoresis and analyzed by immunoblotting. The protein was revealed by an antibody against Gap1 [27]. [file 1475-2859-12-129-S3.pptx]
